# mRNA-Based Vaccines Are Highly Immunogenic and Confer Protection in the Gnotobiotic Pig Model of Human Rotavirus Diarrhea

**DOI:** 10.3390/vaccines12030260

**Published:** 2024-03-01

**Authors:** Casey Hensley, Sandro Roier, Peng Zhou, Sofia Schnur, Charlotte Nyblade, Viviana Parreno, Annie Frazier, Maggie Frazier, Kelsey Kiley, Samantha O’Brien, Yu Liang, Bryan T. Mayer, Ruizhe Wu, Celia Mahoney, Monica M. McNeal, Benjamin Petsch, Susanne Rauch, Lijuan Yuan

**Affiliations:** 1 Department of Biomedical Sciences and Pathobiology, Virginia-Maryland College of Veterinary Medicine, Virginia Tech, Blacksburg, VA 24060, USA; lhcasey@vt.edu (C.H.); pengz81@vt.edu (P.Z.); sofia.schnur@gmail.com (S.S.); charlottejn@vt.edu (C.N.); vparreno2021@vt.edu (V.P.); atf1012@vt.edu (A.F.); mrf1112@vt.edu (M.F.); kelseymk@vt.edu (K.K.); sobrien22@cornellcollege.edu (S.O.); yul20@vt.edu (Y.L.); 2 CureVac SE, 72076 Tübingen, Germany; sandro.roier@curevac.com (S.R.); benjamin.petsch@cipla.com (B.P.); susanne.rauch@curevac.com (S.R.); 3 Vaccine and Infectious Disease Division, Fred Hutchinson Cancer Center, Seattle, WA 98109, USA; bmayer@fredhutch.org (B.T.M.); ruizhw19@gmail.com (R.W.); celiarosemahoney@gmail.com (C.M.); 4 Department of Pediatrics, University of Cincinnati College of Medicine, and Division of Infectious Diseases, Cincinnati Children’s Hospital Medical Center, Cincinnati, OH 45229, USA; mcneal.m12@gmail.com

**Keywords:** rotavirus, mRNA vaccine, gnotobiotic pigs, P2-VP8*, diarrhea

## Abstract

Human rotavirus (HRV) is still a leading cause of severe dehydrating gastroenteritis globally, particularly in infants and children. Previously, we demonstrated the immunogenicity of mRNA-based HRV vaccine candidates expressing the viral spike protein VP8* in rodent models. In the present study, we assessed the immunogenicity and protective efficacy of two mRNA-based HRV trivalent vaccine candidates, encoding VP8* of the genotypes P[8], P[6], or P[4], in the gnotobiotic (Gn) pig model of Wa (G1P[8]) HRV infection and diarrhea. Vaccines either encoded VP8* alone fused to the universal T-cell epitope P2 (P2-VP8*) or expressed P2-VP8* as a fusion protein with lumazine synthase (LS-P2-VP8*) to allow the formation and secretion of protein particles that present VP8* on their surface. Gn pigs were randomly assigned into groups and immunized three times with either P2-VP8* (30 µg) or LS-P2-VP8* (30 µg or 12 µg). A trivalent alum-adjuvanted P2-VP8* protein vaccine or an LNP-formulated irrelevant mRNA vaccine served as the positive and negative control, respectively. Upon challenge with virulent Wa HRV, a significantly shortened duration and decreased severity of diarrhea and significant protection from virus shedding was induced by both mRNA vaccine candidates compared to the negative control. Both LS-P2-VP8* doses induced significantly higher VP8*-specific IgG antibody titers in the serum after immunizations than the negative as well as the protein control. The P[8] VP8*-specific IgG antibody-secreting cells in the ileum, spleen, and blood seven days post-challenge, as well as VP8*-specific IFN-γ-producing T-cell numbers increased in all three mRNA-vaccinated pig groups compared to the negative control. Overall, there was a clear tendency towards improved responses in LS-P2-VP8* compared to the P2-VP8*mRNA vaccine. The demonstrated strong humoral immune responses, priming for effector T cells, and the significant reduction of viral shedding and duration of diarrhea in Gn pigs provide a promising proof of concept and may provide guidance for the further development of mRNA-based rotavirus vaccines.

## 1. Introduction

Human rotavirus (HRV) is the leading cause of childhood viral gastroenteritis, leading to significant morbidity and mortality, primarily in low- and middle-income countries (LMICs) [1,2]. The inclusion of live-attenuated oral vaccines in national vaccination programs has been recommended by the World Health Organization (WHO) since 2006, but their efficacy in LMICs has remained low [2]. Several factors are thought to be responsible, including circulating maternal antibodies, the concurrent use of other oral vaccines, concurrent enteric viral infection, gut dysbiosis, and chronic malnutrition [1,2]. Along with these factors, oral HRV vaccines come with a history of the increased risk of intussusception, a rare but potentially fatal bowel blockage [2]. To circumvent these factors that are mainly associated with the gastrointestinal (GI) tract, other routes of administration have continued to be pursued [2,3]. Intramuscular (IM) non-replicating rotavirus vaccines (NRRV) may offer a solution [4]. The non-replicating nature of these vaccines makes them a safer alternative to live-attenuated vaccines regarding HRV-vaccine-specific intussusception and live-attenuated vaccination risks in general [2,5].

To address the obvious need for improved HRV vaccines in LMICs, CureVac has developed an mRNA-based RNActive^®^ NRRV for intramuscular administration. mRNA as a therapeutic molecule has been studied for decades, but, until recently, there were no licensed mRNA-based vaccines available [6]. mRNA-based vaccines have become widely known in the context of the COVID-19 pandemic, and two are now FDA-licensed for use in the United States [7].

Vaccines employed in this study consist of chemically unmodified, sequence-engineered mRNA featuring optimized non-coding regions previously described in the context of SARS-CoV-2 vaccine development [8,9]. mRNAs are encapsulated in lipid nanoparticles (LNPs) and encode for VP8*, a proteolytic cleavage product of the rotavirus spike protein VP4 that contains neutralizing epitopes and defines the P genotype [10]. Two different mRNA designs, described in more detail in Roier et al. [11], were tested. Both mRNA vaccine designs employed here are trivalent and encode for VP8* derived from the P[4], P[6], and P[8] genotypes. The first design, P2-VP8*, features the universal T-cell epitope P2 fused to VP8* and mirrors a clinically tested protein vaccine [12,13,14]. In LS-P2-VP8*, the second design, P2-VP8* is fused to a multimerization domain derived from *Aquifex aeolicus* lumazine synthase (LS) [15]. The expression of VP8* in this context leads to nanoparticle formation aimed at supporting the formation of 60 m nanoparticles that mimic the natural display of VP8* on the viral surface [11]. The inclusion of LS in other vaccines has been shown to facilitate a more efficient and natural antigen presentation and B-cell activation [16,17,18,19].

The objective of this study was to evaluate the immunogenicity and protective efficacy of these mRNA vaccine candidates in the gnotobiotic (Gn) pig model of HRV infection and diarrhea using the trivalent alum-adjuvanted P2-VP8* protein vaccine, kindly provided by PATH, as a comparator. The G1P[8] Wa HRV Gn pig model is a well-established model that has been used in HRV vaccine evaluation for more than 27 years [20]. It is the only laboratory animal that develops HRV infection, including mimicking human intestinal pathology, and reliably shows clinical signs of disease (diarrhea) [20]. Along with this, the Gn pig mirrors human immunity to HRV and vaccines, with serum IgA, IgG, intestinal IgA, and HRV-specific IFN-γ-producing T cells correlating to protection from HRV infection and diarrhea [20,21]. In general, pigs are physiologically and immunologically similar to humans, making results using swine directly translatable to humans [20,22,23]. Furthermore, the Gn pig lacks any microbiota and the porcine placenta prevents the passive transfer of maternal antibodies. Therefore, Gn piglets are immunologically naïve but immunocompetent at birth [24]. This prevents immune interference from commensal bacteria and maternal antibodies but allows for normal immunological development in response to vaccines [20]. Through this study, we addressed two major questions: (1) Are mRNA vaccines able to confer protection in the Gn pig model of HRV infection and how does their efficacy compare to a protein-based comparator? and (2) Which of the two mRNA vaccine candidates, P2-VP8* or LS-P2-VP8*, confers better protection and immunogenicity in this model?

## 2. Materials and Methods

### 2.1. Rotaviruses for Challenge and Immunoassays

The virulent Wa HRV (G1P[8]) inoculum consisted of a pool composed of small intestinal contents collected from the 28th or 29th passage of the virus in Gn pigs. A dose of 10^5^ focus-forming units (FFU) diluted in 5 mL of Diluent #5 (minimal essential media [Thermo Fisher Scientific, Waltham, MA, USA]; 100 IU of penicillin per mL, 0.1 mg of dihydrostreptomycin per mL; and 1% HEPES) was used for the oral challenge of Gn pigs. The median infectious and diarrhea doses of Wa HRV were determined previously to be approximately 1 FFU [25]. The supernatant of Wa HRV-infected African green monkey kidney MA104 cells (ATCC CRL-2378.1™) were semi-purified by centrifugation through 40% (wt/vol) sucrose cushion and used as a positive control in antigen enzyme-linked immunosorbent assay (ELISA) and cell culture immunofluorescence (CCIF) [26]. Recombinant P genotype-specific P2-VP8* proteins were used as detector antigens in ELISA and ELISpot and as stimulating antigens in flow cytometry analysis of T-cell responses.

### 2.2. mRNA and Protein Vaccines

The trivalent lipid nanoparticle (LNP)-formulated mRNA vaccines P2-VP8* and LS-P2-VP8* were recently described by Roier et al. [11]. The mRNA vaccines are based on CureVac’s RNActive^®^ platform and do not contain chemically modified nucleotides. They have been described in Roth et al. and Gebre et al. [8,9] and comprise a 5′ cap1 structure, a 5′ untranslated region (UTR) from the human hydroxysteroid 17-beta dehydrogenase 4 gene (*HSD17B4*), a GC-enriched open reading frame, a 3′ UTR from the human proteasome 20S subunit beta 3 gene (*PSMB3*), a histone stem-loop, and a poly(A) tail. Production of synthetic mRNA has been described previously [6,27,28]. In short, the open reading frame sequence was generated via gene synthesis and cloned into a basic mRNA vector comprising relevant mRNA elements such as T7 RNA polymerase promotor sequence, 5’-UTR, 3’-UTR, and poly(A) tail. The resulting plasmid DNA (pDNA) was amplified in *E. coli*, purified, sequence-verified, and linearized with restriction enzyme digest. RNA synthesis was carried out by in vitro transcription (IVT) using linearized pDNA as template and co-transcriptional capping. After DNAse digest and cleanup, the IVT product was purified via preparative HPLC, resulting in the final mRNA product. mRNA size and integrity of the final mRNA product was determined using a 12-capillary Fragment Analyzer and PROSize^™^ 3.0 software from Advanced Analytical (Supplementary Figure S1). LNP encapsulation of mRNA was performed using LNP technology from Acuitas Therapeutics (Vancouver, BC, Canada). LNPs are composed of an ionizable amino lipid, phospholipid, cholesterol, and a PEGylated lipid. The P2-VP8* mRNA vaccine design encodes a universal CD4+ T-cell epitope (P2) derived from tetanus toxin [14] (aa residues 830-844; NCBI reference sequence: WP_011100836.1) N-terminally linked to a P genotype-specific rotavirus VP8* subunit protein (aa 65-223 of VP4) by a GSGSG linker. The LS-P2-VP8* mRNA vaccine design encodes full-length lumazine synthase (NCBI reference sequence: WP_010880027.1) from *Aquifex aeolicus* containing two point mutations (C37A to remove an unpaired cysteine and N102D to remove a glycosylation site [16,29]) N-terminally linked via (GGS)_4_-GGG to P2-VP8*, which is designed as described above, except that a longer version of VP8* (aa 41-223 of VP4) is encoded. The P genotype-specific VP8* sequences always derive from the following human rotavirus A strains: BE1128 for P[8] VP4 genotype (GenBank accession number: JN849135.1), BE1322/F01322 for P[6] VP4 genotype (GenBank accession number: JF460826.1), and BE1058 for P[4] VP4 genotype (GenBank accession number: JN849123.1). Protein expression of all constructs was verified in cell culture, expression analyses are described in Roier et al. [11]. The trivalent mRNA vaccines encoding genotypes P[8], P[6], and P[4] of VP8* were formulated by mixing the respective mRNAs in a 1:1:1 weight ratio prior to LNP encapsulation. An LNP-formulated mRNA encoding rabies virus glycoprotein (RABV-G) served as an irrelevant mRNA vaccine and, thus, as a negative control in the present study [30,31].

The recombinant fusion proteins P2-VP8* P[8], P2-VP8* P[6], and P2-VP8* P[4] expressed in *Escherichia coli* were kindly provided by PATH (Seattle, WA, USA) and consist of tetanus toxin T-cell epitope P2 linked to aa 65-223 (for P[8]) or aa 64-223 (for P[6] and P[4]) of VP4 from rotavirus strain Wa (P[8]), 1076 (P[6]), or DS-1 (P[4]), respectively, using a GSGSG linker as previously described [12,13,32,33,34,35]. The protein vaccines were alum-adjuvanted by adsorption of the recombinant fusion proteins to aluminum hydroxide (Alhydrogel^®^; Croda, Frederikssund, Denmark) essentially as previously described [34,36]. Briefly, a two-fold working stock containing 720 µg/mL of trivalent P2-VP8* antigens (1:1:1 weight ratio; 240 µg/mL per antigen) in 0.5 mM PO_4_ in 0.9% saline was combined with an equal volume of an Alhydrogel^®^ suspension (4.48 mg Al/mL diluted in 0.5 mM PO_4_ in 0.9% saline) using gentle mixing for 20–24 h to achieve a formulation with 360 µg/mL total trivalent P2-VP8* antigen containing aluminum hydroxide at 2.24 mg Al/mL in 0.5 mM phosphate buffer in 0.9% saline (pH 6.9). Trivalent P2-VP8* antigen adsorption to Alhydrogel^®^ was analyzed in a previous study and reported to be >90% for formulations with 360 µg/mL antigen containing aluminum hydroxide at 2.24 mg Al/mL in 0.5 mM phosphate buffer [37].

### 2.3. Vaccine Inoculation, Virus Challenge, and Sample Collection of Gn Pigs

Gnotobiotic pigs used in this study were derived via hysterectomy of near-term sows and maintained in germ-free isolators for the duration of the study [38]. Sterility was verified by weekly rectal swabs (RS) on blood agar and in thioglycolate broth. Pigs were fed ultra-high temperature (UHT)-treated sterile whole cow’s milk (The Hershey Company, Hershey, PA, USA) for the duration of the study. On each vaccination day, pigs were injected IM into *M. brachiocephalicus* (PID 0) or left (PID 14) or right (PID 28) *M. biceps femoris* with 30 µg of irrelevant mRNA vaccine (100 µL), with 90 µg (560 µg Al) of P2-VP8* protein vaccine (250 µL), with 30 µg of P2-VP8* mRNA vaccine (100 µL), or with 30 µg or 12 µg of LS-P2-VP8* mRNA vaccine (100 µL each). The first dose of the respective vaccine was administered on post-partum day (PPD) 5 (also referred to as post-inoculation day [PID] 0), followed by two more doses at 14-day intervals for a total of three vaccinations. Serum was collected 14 h after prime vaccination on PID 1, for evaluation of vaccine-induced IFN-α levels. Serum was also collected before each vaccination at PID 14 and 28, before challenge (PID 35 or post-challenge day [PCD] 0), and at euthanasia (PCD 7) to evaluate P2-VP8*-specific IgG and IgA by direct ELISA and virus neutralizing (VN) titers by virus neutralization assay (Supplementary Figure S2). Whole blood was taken on PID 32 for extraction of mononuclear cells (MNCs) for detection of P2-VP8*-specific IFN-γ-producing CD4+ and CD8+ T cells by flow cytometry.

A total of 49 pigs were assigned randomly to five groups (Table 1). Pigs were orally challenged on PID 35 (PCD 0) with 1 × 10^5^ FFU of virulent Wa HRV and monitored for diarrhea and virus shedding via daily RS for PCD 0–7; RS were processed as described previously [26]. All pigs were fed 200 mM sodium bicarbonate 10 min prior to challenge to neutralize stomach acidity. Pigs were euthanized at PCD 7. At euthanasia, small intestinal contents (SIC) and large intestinal contents (LIC) were collected and processed as previously described, for the detection of intestinal antibody responses and antigen presence by direct and sandwich ELISA, respectively, as well as infectious virus particle counting by CCIF [26]. Spleen, ileum, and whole blood were collected for extraction of MNCs to be used in the detection of VP8*-specific IgG and IgA ASC responses by ELISpot and P2-VP8*-specific IFN-γ-producing CD4+ and CD8+ T cells by flow cytometry [39,40].

### 2.4. Assessment of Diarrhea and Detection of Fecal Virus Shedding by Antigen ELISA and CCIF

To assess severity of diarrhea, fecal consistency was recorded, using the RS taken from PCD 0 to 7, as 0: solid, 1: pasty, 2: semi-liquid, and 3: liquid, with any score ≥ 2 being considered diarrhea [26]. Antigen ELISA was used for detection of HRV VP6 and CCIF for detection of infectious virus particles, as previously described [26,41].

### 2.5. Detection of Interferon-α Induction after Prime Immunization

Serum was taken from all pigs 14 h after the prime immunization (PID 1) and interferon-α (IFN-α) levels were evaluated using a commercial ELISA kit (Thermo Fisher cat#ES7RB) according to manufacturer’s instructions. The IFN-α concentrations were calculated based on the standards in the kit and expressed as pg/mL.

### 2.6. Detection of P2-VP8*-Specific Serum and Intestinal IgA and IgG Antibody by ELISA

Serotype-specific anti-P2-VP8* IgA and IgG antibody titers in serum (collected at PID 0, 14, and 28, and PID 35/PCD 0 and PCD 7) and intestinal contents (collected at PCD 7) were measured by using a serotype-specific antibody ELISA [26]. Recombinant P[8], P[6], or P[4] P2-VP8* proteins were used as detector antigens with the coating concentration of 0.5 µg/mL. Negative control wells were coated with coating buffer only (0.05 M carbonate buffer, pH 9.6). Coated plates were incubated for 1 h at 37 °C, washed five times with TBST, and blocked with TBS + 5% BSA overnight at 4 °C. Plates are washed five times with TBST and incubated with four-fold serial dilutions of each serum sample (starting from 1:4 to 1:65,536) overnight at 4 °C. For detection, plates were incubated with goat anti-pig IgA antibody horseradish peroxidase (HRP)-conjugated and goat anti-pig IgG-Fc antibody HRP-conjugated (Bethyl Laboratories, Inc., Montgomery, TX, USA), 1:3000 diluted in TBST with 5% BSA, and developed with ABTS peroxidase substrate (1:1 ratio of KPL ABTS^®^ peroxidase substrate solution A and KPL peroxidase substrate solution B from Seracare Life Sciences, Inc., Milford, MA, USA). The reaction was stopped with ABST peroxidase stop solution diluted 1:5 in ddH_2_O. Optical density was read at 405 nm. IgA and IgG titers were expressed as the reciprocal of the highest sample dilution in which the mean A_405_ of P2-VP8*-coated wells was greater than the mean A_405_ of negative controls plus 3-fold SD. Titers of <4 were assigned a value of 2 for calculation of geometric mean titer (GMT).

### 2.7. Flow Cytometry for Detection of IFN-γ-Producing CD3+CD4+ and CD3+CD8+ T Cells

Mononuclear cells isolated from peripheral blood (PCD 0 and 7) were diluted to a concentration of 1 × 10^6^ cells/mL and seeded into 12-well plates [39]. Cells were re-stimulated for 17 h at 37 °C in 5% CO_2_ with one of six stimulants: (1) medium (negative control), (2) PHA (10 µg/mL; positive control), (3) P[4] P2-VP8* protein (12 µg/mL), (4) P[6] P2-VP8* protein (12 µg/mL), (5) P[8] P2-VP8* protein (12 µg/mL), and (6) semi-purified attenuated Wa HRV antigen (12 µg/mL). Anti-CD49d mAb (0.5 µg/mL) was added to all samples before incubation for co-stimulation. After 12 h, Brefeldin A (5 µg/mL) was added for 5 h at 37 °C in 5% CO_2_. After the total 17 h incubation, cells were washed with 2 mL of commercial stain buffer and transferred to 5 mL Falcon round-bottom polypropylene tubes for 8 min centrifugation at 800× *g* at 4 °C, followed by discarding of the supernatant. Primary antibodies used for staining were mixed in a cocktail that included FITC-conjugated mouse (IgG2b) anti-pig CD4a, SPRD-conjugated mouse (IgG2a) anti-pig CD8a, and mouse (IgG1) anti-pig CD3ε in 100 µL of stain buffer per sample. Samples were incubated with the cocktail for 15 min at 4 °C, and subsequently washed with 500 µL of wash buffer, followed by 8 min centrifugation at 800× *g* at 4 °C. Then, secondary antibody APC-conjugated rat (IgG1) anti-mouse for CD3ε diluted in 100 µL of stain buffer was added and incubated for another 15 min at 4 °C. The washing and centrifugation steps were repeated; then, 100 µL of Permeabilizing/Fixation solution was added into each sample and incubated for 30 min at 4 °C. Washing and centrifugation were then repeated. PE-conjugated mouse (IgG1) anti-pig IFN-γ diluted in 100 µL of stain buffer was added and incubated for another 30 min at 4 °C. A last washing step with 2 mL of stain buffer was performed, followed by centrifugation. Cells were resuspended in 250 µL of stain buffer and stored away from light at 4 °C until delivery to Virginia Tech’s flow cytometry core for acquisition on a BD FACSArial^™^ II flow cytometer.

### 2.8. Detection of P2-VP8*-Specific Antibody-Secreting Cells by ELISpot Assay

Plates (96-well) were coated with 50 µL P[8] P2-VP8* (1.41 mg/mL), P[6] P2-VP8* (1.16 mg/mL), or P[4] P2-VP8* (1.04 mg/mL) proteins diluted to the working concentration of 5 µg/mL in 50 mM carbonate coating buffer (pH 9.6). Plates were incubated at 37 °C for 30 min, then stored at 4 °C overnight. Plates were washed twice with PBST (pH 7.4, with 0.05% Tween 20), blocked with 100 µL/well 4% BSA in PBS (pH 7.4) and incubated at 37 °C for 1 h. Plates were washed three times with ddH_2_0. Separately, extracted MNCs were diluted to a single-cell suspension at a concentration of 5 × 10^6^ cells/mL with E-RPMI, and 100 µL of each diluted cell suspension was added to duplicate wells. Plates were then centrifuged at 500 rpm for 5 min at RT, followed by incubation at 37 °C for 12 h. Plates were then washed five times with PBST, and 100 µL/well of biotinylated goat anti-porcine IgA diluted 1:20,000 (Bethyl A100-102B, 1 mg/mL) or IgG (Bethyl A100-104B, 1 mg/mL) diluted 1:20,000 in PBST was added to each well. Plates were incubated at RT for 2 h and washed five times with PBST. To ensure MNCs were removed completely, the plates were knocked vigorously on paper towels between each wash. HRP-conjugated streptavidin diluted 1:30,000 in PBS was added (100 µL/well) and the plates were incubated at RT for 1 h. Plates were washed five times with PBST, and 50 µL/well KPL TrueBlue HRP substrate (ready-to-use substrate, VWR catalog number 95095-168) was added to each well. Plates were then incubated for 1–2 h at RT until spots become dark blue. Spots were counted with ELISpot analyzer S5 and reported as IgA or IgG ASC per 5 × 10^5^ MNC.

### 2.9. Virus Neutralization Assay

The rotaviruses Wa (G1P[8]), 1076 (G2P[6]), and DS-1 (G2P[4]) were used in this assay. These viruses were originally obtained from the National Institutes of Health and a stock was grown for use in the assay. The rotavirus-neutralizing antibody assay was performed by titrating respective serum samples and incubating them with a constant amount of a specific strain of rotavirus to allow neutralization of the virus. The rotavirus/serum mix was then incubated on MA104 cells in 96-well plates for 1 h to allow adsorption of the virus. The plates were washed to remove serum, and then overlaid with media. Control wells on each 96-well plate received either diluted virus without sera (virus control) or no virus or sera (cell controls). After an overnight incubation allowing for rotavirus growth, the cells were frozen and thawed to lyse the cells to release the viruses, and then the lysates were resuspended. The relative amount of non-neutralized rotavirus was determined using an antigen-capture ELISA to detect rotavirus. The ELISA plates were coated with rabbit anti-rotavirus IgG antibodies. The resuspended lysates from the neutralization part of the assay were added and incubated. Following blocking with nonfat milk, guinea pig anti-rotavirus antiserum was added to the wells to detect captured rotavirus. Horseradish peroxidase (HRP)-conjugated rabbit anti-guinea pig IgG (Jackson ImmunoResearch, West Grove, PA, USA) was added to detect the guinea pig anti-rotavirus antibody. The wells were developed with o-Phenylenediamine (Sigma-Aldrich, St. Louis, MA, USA), a peroxidase-sensitive colorimetric substrate. Optical density of each well was read as absorbance at 492 nm. The amount of rotavirus present in the resuspended lysate from each well is inversely related to the amount of neutralizing antibody present in the serum. Each serum dilution series was modeled using a logistic regression function. For each fitted curve, the reciprocal serum dilution which corresponds to a 40% response (ED_40_), compared to the virus controls, was determined and reported as the titer. The ED_40_ represents the titer of the serum against a given virus, which represents a 60% reduction in the amount of virus [42].

### 2.10. Statistical Analysis

Pigs were randomly assigned to treatment groups by animal care staff upon derivation, regardless of sex or size. Proportions of affected animals (infected and with diarrhea) among treatment groups were compared using Fisher’s exact test.

Daily mean fecal consistency (diarrhea) scores from PCD 0 to PCD 7 among treatment groups were analyzed using a two-way ANOVA of repeated measures through time, followed by Holm–Šídák’s multiple comparisons test. Daily mean CCIF and ELISA titers of virus shedding as independent variables were also analyzed by a two-way ANOVA of repeated measures through time, followed by Tukey’s multiple comparisons test.

CCIF titers and fecal consistency scores were summarized over time for each animal into six efficacy endpoints: mean peak titer (maximum CCIF titer), the area under the curve over time (AUC) of virus shedding, AUC of fecal scores, cumulative fecal consistency score, the onset and duration of viral shedding (total days of detectable titers), and the onset and duration of diarrhea (total days with fecal consistency scores > 1). The area under the curve (AUC) of diarrhea score and virus shedding were calculated in RStudio using the AUC command of the DescTools package and the spline approach. Peak titer, AUC of virus shedding, AUC of diarrhea, and cumulative fecal score were analyzed using a fitted linear mixed effects model to assess the vaccine group effect on each endpoint with litter random intercept, followed by pairwise *t*-tests using the Kenward–Roger method [43] and exact one-step multiplicity adjustment. Duration of viral shedding and diarrhea were analyzed using the Prentice rank-sum test with litter specified as a block variable, followed by Wilcoxon rank-sum tests with Holm’s multiplicity correction. For comparing intestinal ELISA antibody and serum VN antibody titers, the titers were log-transformed before statistical analysis. The mean antibody titers among groups were analyzed by a general mixed model of repeated measures through time. Total numbers and mean frequencies of T-cell subsets were analyzed using ordinary one-way ANOVA, followed by Dunnett’s multiple comparisons test. Mean numbers of ASCs in post mortem tissues were analyzed using the Kruskal–Wallis test, followed by Dunn’s multiple comparisons test. P-type specific serum IgG and IgA responses were analyzed using the Prentice rank-sum test with litter specified as a block variable, followed by Wilcoxon rank-sum tests with Holm’s multiplicity correction. IFN-α levels were analyzed using a mixed effects model with litter random intercept, followed by pairwise t-tests using the Kenward–Roger method and exact one-step multiplicity adjustment. Spearman correlations were tested within efficacy endpoints and between efficacy and immunogenicity endpoints.

A sensitivity analysis was also performed to determine the influence of litter variation on the efficacy results. For the efficacy endpoints analyzed via the mixed models, two variance components were estimated: the intra-experiment variability (i.e., litter to litter variation via the litter random effect) and the residual variance (i.e., the measurement error). For duration of viral shedding and diarrhea, these variance components were extracted from a one-way ANOVA with litter as the independent variable. Using these components, the intraclass correlation coefficient (ICC) for litter variation was computed: the ratio of litter variance over the total variance. The ICC ranges from 0% to 100%, where 0% indicates that the experimental variability is entirely attributable to residual variation (i.e., there is no meaningful litter to litter variation) and 100% indicates that the experimental variability is entirely attributable to litter variation. The ICC for all six endpoints was minimal (<8%), suggesting that the litter variability explained little result variation between groups.

Statistical analyses were performed using GraphPad Prism 9 (GraphPad Software, San Diego, CA, USA) and R (R Core Team, Vienna, Austria). A *p*-value lower than 0.05 was considered as statistically significant. In cases where unadjusted p-values were statistically significant but the adjusted *p*-values were not, the unadjusted significance was reported.

## 3. Results

### 3.1. mRNA Vaccines Induce IFN-α in Serum of Vaccinated Animals 14 h Post Immunization

Serum collected 14 h post-prime immunization was evaluated for the systemic induction of IFN-α. As expected, the lowest levels of IFN-α with a mean value of 167 pg/mL were detected in animals vaccinated with the P2-VP8* protein vaccine. All mRNA vaccines induced IFN-α with the lowest mean levels of 237 pg/mL detectable for LS-P2-VP8* at a dose of 12 µg, and 30 µg of P2-VP8* and LS-P2-VP8* induced approximately two-fold higher levels with mean IFN-*α* values of 557 and 442 pg/mL, respectively (Supplementary Figure S3).

### 3.2. Both mRNA Vaccine Candidates Conferred Significant Reduction of Severity and Duration of Diarrhea, and Elicited Significant Protection against Infectious Virus Shedding

**Figure 1:**
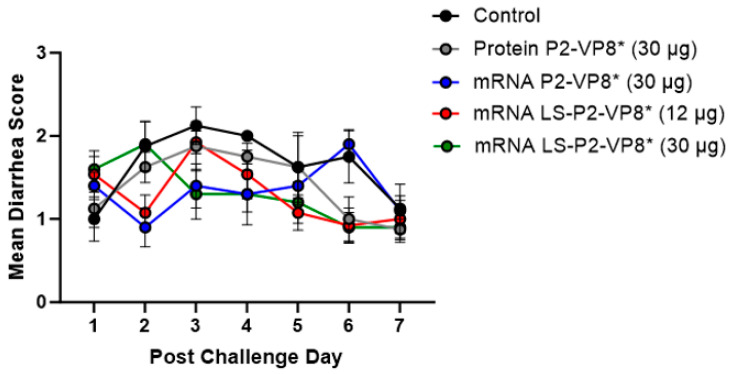
Daily mean diarrhea scores for PCD 0–7 in vaccinated and control pigs. Daily rectal swabs were taken for evaluating fecal consistency scores after challenge with Wa HRV. There were no significant differences between groups at any timepoint using two-way ANOVA followed by Holm–Šídák’s multiple comparisons test (*n* = 8–13). Circles indicate means with SEM.

Gn pigs were challenged with virulent Wa HRV at post-inoculation day (PID) 35/post-challenge day (PCD) 0, and clinical signs and virus shedding was monitored via daily rectal swabs (RS). These data are summarized in Table 2. The percentage of animals with diarrhea and the mean days to onset remained largely unchanged compared to negative control animals for all mRNA, as well as the protein comparator groups. Pigs who received mRNA P2-VP8* (30 µg) and LS-P2-VP8* (30 µg) had a significantly shortened duration of diarrhea compared to the negative control pigs (3.1 days and 2.5 days versus 4.6 days, respectively) (Table 2). All pigs who received mRNA vaccines had a significantly decreased AUC of diarrhea that was 2.2–2.6 lower than control pigs (Table 2). Indeed, negative control pigs experienced a double peak of diarrhea at PCDs 3 and 6, while pigs vaccinated with LS-P2-VP8* (12 µg and 30 µg) only experienced one peak at PCD 2 and PCD 3, respectively, which then tapered off to consistently lower scores for the rest of the study (Figure 1). mRNA P2-VP8* (30 µg)-vaccinated pigs experienced a slight peak at PCD 3, and a larger peak, comparable to control pigs, at PCD 6 (Figure 1). Throughout the observation period, mean diarrhea score values were overall lower in all mRNA groups compared to the negative control with the exception of PCD 6 for the P2-VP8* group, where scores were comparable. The reduced severity of diarrhea was also reflected by significantly decreased cumulative fecal scores that were 2.1–2.4 lower in the mRNA vaccine groups compared to the controls (Table 2). Protein P2-VP8*-vaccinated pigs experienced overall similar trends to the mRNA groups. However, there was a trend towards improved values for LS-P2-VP8* mRNA vaccine compared to protein P2-VP8* in terms of the mean duration days (30 µg only), mean cumulative fecal score, and AUC of diarrhea (both dose groups). In addition, mean diarrhea scores after the peak at PCD 3 trended higher in protein than in mRNA-vaccinated groups for the duration of the study. An exception was PCD 6, where mean diarrhea scores in the protein P2-VP8* group were comparable to mRNA LS-P2-VP8* vaccines but were lower than in mRNA P2-VP8*-vaccinated pigs. (Figure 1). Overall, a trend towards improved clinical signs elicited by LS-P2-VP8* compared to the mRNA P2-VP8* and the protein P2-VP8* was detected.

**Figure 2:**
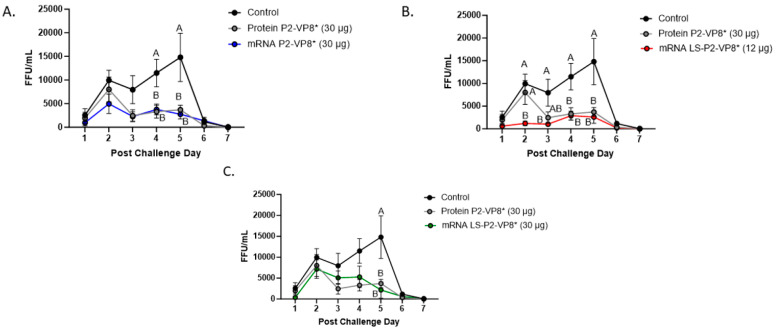
Daily fecal virus shedding as measured by CCIF for PCD 0–7 in mRNA P2-VP8* (30 μg) (**A**), mRNA LS-P2-VP8* (12 μg) (**B**), and mRNA LS-P2-VP8* (30 μg)-vaccinated pigs (**C**) versus control and protein P2-VP8* (30 μg)-vaccinated pigs. Daily rectal swabs were taken for evaluating fecal virus shedding after challenge with Wa HRV. Fecal infectious virus particles were measured by CCIF and results are expressed as FFU/mL. Fecal samples from mock-infected pigs were used as negative controls. Different capital letters above data points indicate significant differences (adjusted *p* < 0.05) between groups at the same time point, while shared letters or no letters indicate no significant difference, according to two-way ANOVA, followed by Tukey’s multiple comparisons test. Circles indicate means with SEM. CCIF, cell culture immunofluorescence; FFU, focus forming units.

The mean duration days of virus shedding was significantly reduced (to 3.6–4.1 days) in all pigs who received mRNA vaccines compared to the control group (5.4 days) (Table 2). The AUC of virus shedding was also significantly reduced in all pigs who received mRNA vaccines, ranging from 2.2-fold to 5.7-fold lower than the control (Table 2). Similar to the diarrhea data, when looking at mean daily titers, negative control and protein P2-VP8*-vaccinated pigs experienced a classic double peak of daily mean CCIF titers at PCDs 2 and 5 (Figure 2), although this effect was more pronounced in control pigs [21,22,44,45]. Pigs vaccinated with mRNA P2-VP8* shed a significantly lower amount of virus than negative control pigs on PCDs 4–5 and displayed a double peak that was comparable to the P2-VP8* protein groups (Figure 2A). In contrast, LS-P2-VP8* (12 µg)-vaccinated pigs shed at significantly lower titers compared to negative control pigs on PCDs 2–5 and to P2-VP8* protein at PCD 2 (Figure 2B). Titers remained low throughout the observation period and displayed a single low peak on PCDs 4–5 (Figure 2B). When comparing LS-P2-VP8* (12 µg)-vaccinated pigs to protein-P2-VP8*-vaccinated pigs, this group shed significantly lower titers on PCD 2 (Figure 2B). The results for LS-P2-VP8* (30 µg)-vaccinated pigs were not as pronounced; however, the group’s mean titer around the time of the typical second shedding peak (PCD 5) was significantly lower than the negative-control-vaccinated pigs (Figure 2C). This is reflected by the previously mentioned significantly shortened duration of virus shedding in this group (Table 2). For peak viral titers, the mRNA P2-VP8* and LS-P2-VP8* (12 µg)-vaccinated pigs exhibited significantly reduced (3.0-fold and 4.7-fold lower, respectively) titers compared to negative control pigs. When evaluating mRNA-vaccinated pigs against the protein-P2-VP8* vaccine (negative control excluded in analysis), LS-P2-VP8* (12 µg) conferred significantly higher protection with a 2.3-fold lower AUC of virus shedding, 2.2-fold lower peak titer, and 1.3 days less shedding.

Correlations across six efficacy measures of the rotavirus disease burden (AUC of virus shedding, peak titer, shedding days, AUC of diarrhea, cumulative fecal score, and diarrhea days) were examined (Supplementary Figure S4). The highest Spearman correlations were observed within the virus-shedding and the diarrhea measurements. Cross virus-shedding and diarrhea measurement correlations were uniformly lower but all positive. The positive correlation is consistent with a link between viral titers and symptomatic disease (as measured by fecal consistency scores), but an incomplete correlation indicates that worse diarrhea outcomes are not entirely explained by high viral titers.

### 3.3. LS-P2-VP8* Vaccine Induced Strong P-Type-Specific Serum IgG Antibody Responses Pre- and Post-Challenge and Primed for Stronger Serum IgA Antibody Responses Post-Challenge Compared to Other Groups

**Figure 3:**
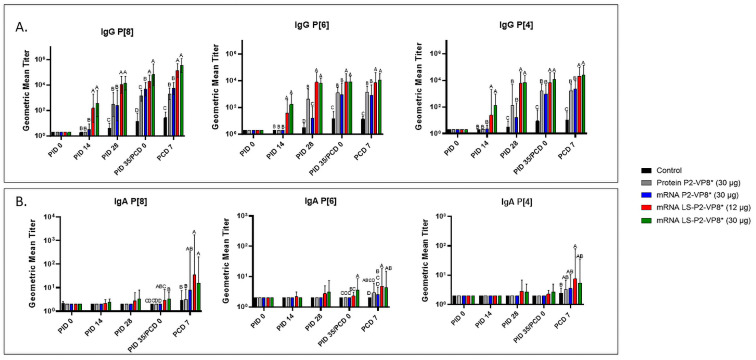
Geometric mean titers of P-type-specific IgG (**A**) and IgA (**B**) antibodies in Gn pig serum samples collected at PID 0, PID 14, PID 28, PID 35/PCD 0, and PCD 7. Samples were tested at a series of 4-fold dilutions, beginning at 1:4. All negative samples were given a titer of 2 to allow for data analysis and use in graphical depictions. Different capital letters above bars indicate significant differences (unadjusted *p* < 0.05) between groups, while shared letters or no letters indicate no significant difference. Bars indicate geometric means with geometric SD.

Serum samples were collected from all pigs before each vaccination (PID 0, 14, and 28), challenge (PID 35/PCD 0), and at euthanasia (PCD 7). P-type-specific IgG and IgA antibody responses were analyzed in the serum at each time point via ELISA, and the results are summarized in Figure 3. Both doses of LS-P2-VP8* induced significantly stronger IgG responses to P[8], P[6], and P[4] in the serum compared to all other groups, beginning at PID 14, after only one immunization (Figure 3A). This trend continued to PCD 7, at which point both dose groups still had significantly higher titers than all other groups for all three P-types.

The correlations of the pre-challenge immune responses (serum IgG and serum IFN-α) with the efficacy measures of the rotavirus disease burden (AUC of virus shedding, shedding days, AUC of diarrhea, and diarrhea days) were assessed. For P-type-specific serum IgG responses at PID 28 and PCD 0, a consistent negative correlation was observed, highlighting that higher IgG responses correlated with lower measures of disease burden (Supplementary Figure S5). At either time point, all but one correlation between IgG response titers and the virus-shedding measurements were significant. While there was uniform negative correlation with the diarrhea measurements, the only significant correlation was between anti-VP8* P[8] IgG titers and the AUC of diarrhea at PID 28 and PCD 0. There were no significant correlations found with serum IFN-α responses induced 14 h post-prime immunization and the efficacy measurements (Supplementary Figure S6).

Low levels of P-type-specific IgA titers were detected in LS-P2-VP8*-vaccinated pigs as early as PID 14, but not to a significant degree until PID 35, when P[8]- and P[6]-specific serum IgA titers were significantly higher in the LS-P2-VP8* (30 µg) groups compared to negative control, protein P2-VP8*, and mRNA P2-VP8*-vaccinated pigs (Figure 3B). Post-challenge, both doses of LS-P2-VP8* vaccine primed for significantly higher serum IgA against P[8] and P[6] VP8* compared to negative control and protein P2-VP8*-vaccinated pigs, with the exception of IgA against P[6], where the increase induced by 30 µg LS-P2-VP8* was not significantly higher than in the protein P2-VP8* group. P[4]-specific IgA titers were significantly higher in LS-P2-VP8* (12 µg)-vaccinated pigs than the negative control pigs post-challenge (Figure 3B).

### 3.4. LS-P2-VP8* Vaccine Primed for Stronger P[8]-Specific Small Intestinal IgA and IgG Antibody Responses Post-Challenge Compared to Other Groups

**Figure 4:**
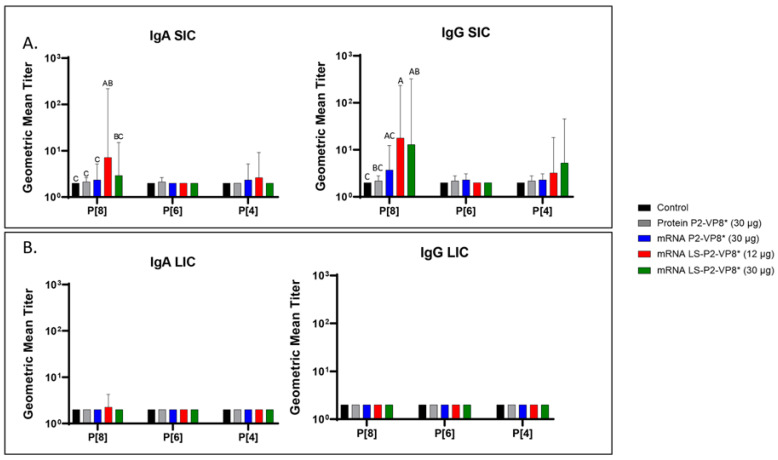
Geometric mean titers of P-type-specific IgG and IgA antibodies in Gn pig intestinal content samples collected at PCD 7. Samples were tested at a series of 4-fold dilutions, beginning at 1:4. All negative samples were given a titer of 2 to allow for data analysis and use in graphical depictions. Different capital letters above bars indicate significant differences (adjusted *p* < 0.05) between groups, while shared letters or no letters indicate no significant difference, according to mixed-effects analysis, followed by Tukey’s multiple comparisons test. (**A**) SIC, small intestinal contents; (**B**) LIC, large intestinal contents. Bars indicate geometric means with geometric SD.

Small and large intestinal contents (SIC and LIC, respectively) were collected at euthanasia (PCD 7) for the quantification of local P-type-specific IgG and IgA responses induced by the vaccine candidates using the same ELISA as was used for serum samples. In the SIC, LS-P2-VP8* (12 µg) vaccine primed for significantly stronger P[8]-specific IgA and IgG responses post-challenge compared to controls and protein P2-VP8*-vaccinated pigs. (Figure 4A). There were some detectable titers of P[4]-specific IgA and IgG in the SIC of pigs vaccinated with both mRNA P2-VP8* and LS-P2-VP8* (12 µg), but not to a significant degree (Figure 4A). This is likely explained by the relatively high sequence homology between the P[8] (challenge strain) and P[4] genotypes [46]. There were no detectable IgG or IgA titers in the LIC at PCD 7, except for nominal P[8]-specific IgA titers in the LS-P2-VP8* (12 μg) group. (Figure 4B).

### 3.5. LS-P2-VP8* (30 µg) Vaccine Induced Stronger HRV-Specific T-Cell Responses in the Blood Pre-Challenge and Primed for Stronger CD4^+^ Responses Post-Challenge Compared to Other Groups

**Figure 5:**
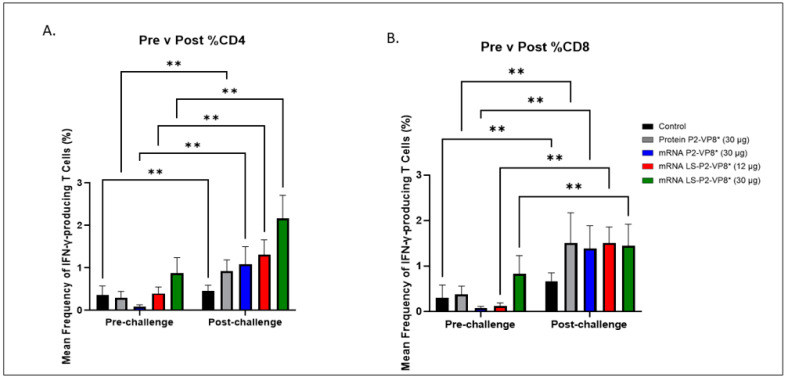
Mean frequencies (%) of CD3+CD4+IFN-γ+ (**A**) and CD3+CD8+IFN-γ+ (**B**) T cells in the blood pre- versus post-challenge. For comparing between pre- and post-challenge for the same group, two-way ANOVA, followed by Tukey’s multiple comparisons test, was used (*n* = 8–13; ** *p* ≤ 0.01). Bars indicate means with SEM.

MNCs were extracted from peripheral blood pre-challenge (PCD 0) and post-challenge (PCD 7), and frequencies and total numbers of CD3^+^CD4^+^IFN-γ^+^ and CD3^+^CD8^+^IFN-γ^+^ T cells among MNCs were measured by flow cytometry and intracellular staining (Supplementary Figure S7). There were no significant differences in the absolute numbers or frequencies among MNCs stimulated with different antigens (P[4], P[6], or P[8] P2-VP8* protein, or attHRV antigen). To simplify the data presentation, we calculated the means of IFN-γ+ T-cell numbers or frequencies from P[4], P[6], P[8] and attHRV antigen-stimulated MNCs. The numbers or frequencies from antigen-stimulated MNCs were subtracted by the numbers or frequencies from mock-stimulated MNCs and assigned as HRV-specific IFN-γ+ T cells from each animal. Mean total numbers and frequencies of CD3+CD4+IFN-γ+ and CD3+CD8+IFN-γ+ T cells among all pigs from the five different treatment groups are presented in Figure 5 and Supplementary Figure S8.

The 30 µg LS-P2-VP8* mRNA vaccine induced a greater pre-challenge T-cell response that primed for a better post-challenge response of both effector and memory cells, presumably. The LS-P2-VP8* mRNA vaccine induced higher numbers (both 12 µg and 30 µg dose) and frequencies (30 µg dose only) of CD3+CD4+IFNγ+ T cells pre-challenge compared to all other groups, although they were not statistically significant (Supplementary Figure S8A). Mean frequencies of CD3+CD8+IFNγ+ pre-challenge in the LS-P2-VP8* (30 µg) group were higher than in all other groups, although, again, they were not statistically significant (Figure 5B). All groups experienced statistically significant increases in HRV-specific mean frequencies of CD3+CD4+IFN-γ+ and CD3+CD8+IFN-γ+ T cells after challenge (Figure 5A,B). LS-P2-VP8* (30 µg) primed for significantly higher post-challenge mean frequencies of HRV-specific CD3+CD4+IFN-γ+ T cells compared to all other groups (Figure 5A). This trend was not detectable for CD8^+^ T cells, where higher mean frequencies post-challenge were induced that were comparable for all vaccinated pigs (Figure 5B).

### 3.6. LS-P2-VP8* Vaccine Primed for Higher Blood and Intestinal ASC Responses Post-Challenge Compared to the Control

**Figure 6:**
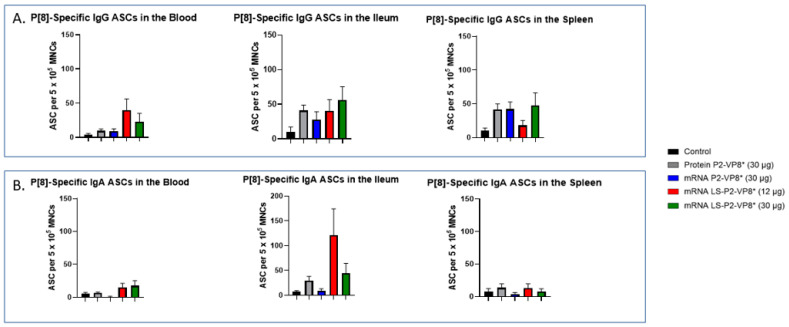
Mean numbers of P[8]-specific ASCs in the tissues of Gn pigs post-challenge. MNCs extracted from post mortem tissues were evaluated for P-type-specific IgG (**A**) or IgA (**B**) ASCs using ELISpot assay. Kruskal–Wallis test, followed by Dunn’s multiple comparisons test, was used for analysis (*n* = 8–13). Bars indicate means with SEM. ELISpot, enzyme-linked immunosorbent spot; ASC, antibody-secreting cells; MNCs, mononuclear cells.

MNCs extracted from the blood, ileum, and spleen were used to evaluate P-type-specific IgG and IgA ASC responses post-challenge by an ELISpot assay. LS-P2-VP8*-vaccinated pigs had higher numbers of P[8]-specific IgG ASCs detected in the blood and exhibited a trend towards increased responses in the ileum (30 µg group) post-challenge compared to other groups, although not to a significant degree. Protein P2-VP8*-vaccinated pigs had numbers comparable to mRNA P2-VP8* in the blood and spleen, and to LS-P2-VP8* (12 μg)-vaccinated pigs in the ileum (Figure 6A). All vaccine groups except for the 12 µg LS-P2-VP8* group induced higher numbers of P[8]-specific IgG ASCs in the spleen compared to the negative control, indicating that vaccination was able to prime for a systemic memory B-cell response upon subsequent virus exposure in the secondary lymphoid tissue (Figure 6A). Similarly, all LS-P2-VP8*-vaccinated pigs were primed for higher numbers of P[8]-specific IgA ASCs in the blood and ileum post-challenge, although these numbers were not statistically significant. (Figure 6B).

The numbers of P[6]-specific IgG ASC in the ileum and spleen were similar across all groups post-challenge (Supplementary Figure S9A). A statistically not significant increase was detected for P[6]-specific IgA ASCs in the ileum upon vaccination with LS-P2-VP8* (12 μg) (Supplementary Figure S9B). Among all groups, both LS-P2-VP8* mRNA vaccine doses induced the highest numbers of P[6]- and P[4]-specific IgA ASCs in the blood post-challenge (Supplementary Figures S9B and S10B), while the 30 µg dose of LS-P2-VP8* induced the highest levels of P[4]-specific IgG ASCs in the ileum (Supplementary Figure S10A). LS-P2-VP8* (12 μg) elicited high mean numbers of P[4]-specific IgA ASCs in the ileum with a high variability (Supplementary Figure S10B).

### 3.7. Pigs Vaccinated with LS-P2-VP8* Were Primed for Significantly Higher P[8]- and P[6]-Specific VN Antibody Titers Post-Challenge Compared to Other Groups

**Figure 7:**
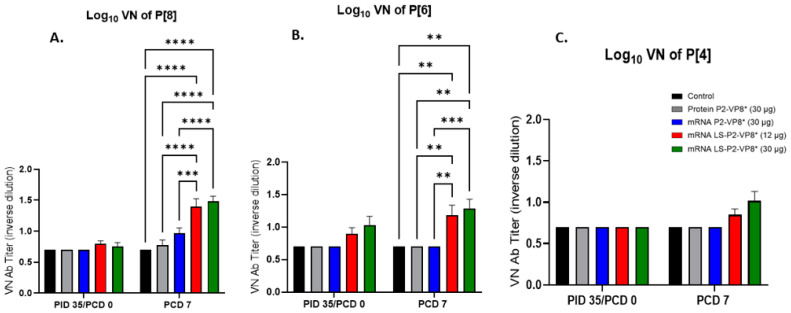
Serum virus neutralizing antibody titers to P[8] (**A**), P[6] (**B**), and P[4] (**C**) HRV in vaccinated Gn pigs pre- and post-challenge. Serum taken prior to each vaccination and challenge was used to evaluate VN antibody titers specific to P[8] (Wa strain), P[6] (1076 strain), and P[4] (DS-1 strain) HRVs. Values less than 10 were set to 5 for statistical analysis. Mixed-effects model, followed by Tukey’s multiple comparisons test, was used for analysis (*n* = 8–13; ** *p* ≤ 0.01, *** *p* ≤ 0.001, **** *p* ≤ 0.0001). Bars indicate means with SEM.

Serum samples collected pre- and post-challenge were used to evaluate P-type-specific neutralizing antibody (nAb) titers in Gn pigs. None of the vaccines induced significant VN titers pre-challenge compared to the negative control group (Figure 7). However, both LS-P2-VP8* doses induced responses against P[4], P[6], and P[8] serotypes on PCD 7, while little or no responses were induced in the other groups. nAb titers against P[8]- and P[6] viruses were significantly higher in LS-P2-VP8*-vaccinated animals compared to both negative control and protein P2-VP8*-vaccinated pigs (Figure 7A,B).

## 4. Discussion

In this study, the immunogenicity and protective efficacy of two mRNA vaccines were evaluated and compared between each other and to the protein P2-VP8* vaccine using the highly relevant Gn pig challenge model. When compared to both the protein P2-VP8* and negative control animals, the two mRNA vaccine candidates conferred improved protection from virus shedding, as evidenced by the reduced duration and daily titers (AUC) measured by CCIF. Despite the significant reduction in virus shedding, particularly in LS-P2-VP8*-vaccinated groups, diarrhea was still seen in almost all pigs. This is not unexpected, as it has been shown that HRV triggers the release of adenosine 5′-diphosphate (ADP) in infected cells, resulting in the dysregulation of calcium signaling in neighboring, uninfected cells [47]. This leads to secretory diarrhea, even in the presence of minimal virus infection of the intestinal epithelial cells [47]. Nevertheless, the LS-P2-VP8* mRNA vaccine at both doses conferred partial protection against diarrhea that was improved compared to protein P2-VP8* and negative-control-vaccinated pigs upon challenge with the virulent Wa HRV. This was evidenced by the significantly reduced mean duration of diarrhea in the LS-P2-VP8* (30 µg) group, as well as the consistently lower mean daily diarrhea scores in both LS-P2-VP8* groups. Along with this, there was one pig each in both LS-P2-VP8* groups that did not experience any diarrhea at any point between PCDs 1–7. We expect, with a larger sample size, that there would be a greater number of LS-P2-VP8*-vaccinated pigs that experience little to no diarrhea. Both doses of the LS-P2-VP8* mRNA vaccine were also highly immunogenic and induced stronger serum IgG antibody responses specific to P[8], P[6], and P[4] HRVs both pre- and post-challenge, as well as stronger serum IgA to all three P-types post-challenge compared to all other groups. These data agree with the rodent model data, which showed that the LS-P2-VP8*-based mRNA vaccines perform better in terms of immunogenicity than those encoding P2-VP8* only [11]. It is conceivable that parenteral vaccine protection will be illustrated by markers, such as HRV-specific serum IgG and IgA [48,49,50,51], despite the historical use of intestinal IgA as a correlate of protection for live oral attenuated vaccines. Evidence supporting this idea includes the fact that hyperimmune serum has been shown to protect nonhuman primates from an RV challenge, as well as the long-observed phenomenon of maternal antibody protection from natural HRV infection in the first few months of life, before vaccination can safely occur [44,52]. Accordingly, the correlation analyses performed in the present study indicate a link between higher serum IgG responses and a lower disease burden.

Furthermore, LS-P2-VP8* vaccines were the only groups tested that were able to prime for low levels of P[8]-specific intestinal IgA and IgG responses post-challenge. Titers in this compartment were likely undetectable and low pre-and post-challenge, respectively, since inducing mucosal IgA through the use of a parenteral vaccine is generally difficult [45]. These low responses may still contribute to protection since local mucosal IgA responses are a historically important correlate of protection in natural HRV infection, as well as with the use of live oral attenuated vaccines [48,49,50]. The immunogenicity seen, especially the P[8]-specific IgA and P[8]-specific IgA ASCs in the intestine, is consistent with the significant protection from virus shedding seen in the LS-P2-VP8* groups.

Along with B cells, CD8^+^ T cells play a vital role in the total clearance of HRV infection and diarrhea in Gn pigs, even in the absence of B cells, as evidenced by a study that showed significant increases in both diarrhea and the virus-shedding severity that persisted past PCD 7 in CD8^+^ T-cell-deficient Gn pigs [53]. Although we did see significant increases in all groups between the pre- and post-challenge timepoints, there was no meaningful difference detectable between vaccinated groups. It is, therefore, reasonable to assume these groups’ post-challenge T-cell populations were largely comprised of primary effector T cells stimulated by the virus challenge itself. Elucidating more specific markers for these populations would prove useful in future studies.

Interestingly, the lower dose (12 µg) of LS-P2-VP8* performed better than the higher dose (30 µg) in terms of protection from clinical symptoms and viral shedding. One reason for this may be the lower stimulation of innate responses such as the IFN-α level induced by LS-P2-VP8* (12 µg). Type I IFNs have been shown to have a double-edged effect in terms of mRNA vaccination. A dramatic systemic response is generally not preferable, as it may increase the side effects of vaccinations such as headache, fever, and fatigue, as well as the possibility of stimulating a diffuse antiviral state, preventing antigen uptake and subsequent translation [54]. However, a mild, local type I IFN response may be associated with stronger downstream adaptive responses, due to the efficient recruitment of APCs to the site [55]. It is important to note, however, that, despite lower IFNα levels being induced by protein P2-VP8* vaccination, these did not correlate with increased immunogenicity as was seen in the LS-P2-VP8* (12 µg)-vaccinated pigs, and there are likely other factors playing a larger role. This is further supported by the fact that we generally found no significant correlations between IFN-α responses and the efficacy measurements.

The protein vaccine P2-VP8* did not confer the same protection as reported in a previous Gn pig study [12] that assessed a monovalent version of the protein vaccine P2-VP8* P[8] in a dose of 50 µg. This dose induced better protection from P[8] Wa HRV-induced diarrhea and virus shedding than the 90 µg dose of trivalent P2-VP8* protein vaccine employed in the present study, that only contains 30 µg of each VP8* antigen (P[8], P[6], and P[4]), indicating a dose effect of the vaccine matching the challenge strain as a possible reason the discrepancy between both studies. The dose of 90 µg represents a full human dose (NCT04010448) and was chosen because its immunogenicity, safety, and efficacy, i.e., ability to reduce fecal shedding of attenuated HRV in humans, were demonstrated in previous studies [12,13,56]. The ongoing Phase 3 clinical trial (NCT04010448) assessing the safety and efficacy of the trivalent P2-VP8* protein vaccine met the futility criteria at the interim analysis, as the protein vaccine demonstrated no superior efficacy to a licensed oral rotavirus vaccine [3,57].

Although the mRNA candidates tested in this study were highly immunogenic and conferred significant protection from virus shedding and diarrhea, it is still unclear how these results of mRNA-based rotavirus vaccines will translate to humans. As shown here and as recently discussed [11], the differences in immune responses elicited by protein and mRNA vaccines support the hypothesis that the mRNA-encoded VP8* is able to elicit improved immune responses over protein-based vaccines and may, therefore, represent a viable approach for testing in a clinical setting. Parenteral vaccines for enteric viruses, especially HRV, remains a subject of further research, and continue to show their status as a promising avenue of exploration for improving HRV vaccines.

## 5. Conclusions

Our study demonstrated the immunogenicity and partial protection induced by two trivalent mRNA vaccine candidates, P2-VP8* and LS-P2-VP8*, against HRV in comparison to a P2-VP8* protein vaccine and an irrelevant mRNA vaccine, using the neonatal Gn pig model.

Both mRNA vaccines performed comparably or better (12 µg LS-P2-VP8*) than the P2-VP8* protein vaccine concerning the reduction of diarrhea and viral shedding. An analysis of humoral and cellular responses demonstrated increased values upon vaccination with LS-P2-VP8* compared to the protein vaccine.

The trivalent LS-P2-VP8* mRNA vaccine, in which VP8* was engineered to be expressed as a secreted nanoparticle, yielded improved responses over the trivalent P2-VP8* mRNA vaccine, which encodes for cytoplasmic, monomeric VP8*. While both vaccines reduced clinical symptoms and conferred significant protection from virus shedding, there was a trend towards improved responses for LS-P2-VP8*. In addition, LS-P2-VP8* mRNA was able to elicit higher levels of humoral and cellular responses than the P2-VP8* mRNA vaccine. Overall, this study provides the proof of principle of an mRNA vaccine against a non-enveloped virus in a highly relevant infection model and may help to lay the groundwork for the further development of a much-needed NRRV.

## Figures and Tables

**Table 1:** Allocation of Gn pigs.

Group	*n*	Vaccine	Challenge
1	8	Irrelevant LNP-formulated mRNA vaccine (**Control**)	Wa HRV (G1P[8])
2	8	Trivalent alum-adjuvanted P2-VP8* protein vaccine (Protein P2-VP8*)	
3	10	Trivalent LNP-formulated P2-VP8* mRNA vaccine (P2-VP8*)	
4	13	Trivalent LNP-formulated LS-P2-VP8* mRNA vaccine (LS-P2-VP8* [12 µg])	
5	10	Trivalent LNP-formulated LS-P2-VP8* mRNA vaccine (LS-P2-VP8* [30 µg])	

**Table 2:** Diarrhea and virus shedding in mRNA-vaccinated and control pigs after challenge with virulent Wa HRV (G1P[8]).

Clinical Signs of Diarrhea ^b^							Virus Shedding (CCIF) ^c^				
Treatments (Vaccine Dose µg) ^a^	*n*	Percentage with Diarrhea	Mean Days to Onset	Mean Duration Days	Mean Cumulative Fecal Score	AUC of Diarrhea	Percentage of Shedding Virus	Mean Days to Onset	Mean Duration Days	Mean Peak Titer (FFU/mL of Feces) ^f^	AUC of Virus Shedding
**Control**	8	8/8 (100%)	2	4.6 (0.53) ^Ade^	11.5 (0.78) ^A^	10.4 (0.65) ^A^	8/8 (100%)	1.5	5.4 (0.18) ^A^	2.1 × 10^4^ (3645) ^A^	4.7 × 10^4^ (6881) ^A^
Protein P2-VP8* **(30 µg)**	8	8/8 (100%)	1.9	3.1 (0.35) ^AB^	9.9 (0.74) ^AB^	8.9 (0.78) ^AB^	8/8 (100%)	1.3	4.9 (0.30) ^AB^	1.0 × 10^4^ (2182) ^ABC^	1.9 × 10^4^ (4030) ^AB^
mRNA P2-VP8* **(30 µg)**	10	10/10 (100%)	2.4	3.1 (0.10) ^B^	9.4 (0.48) ^AB^	8.2 (0.48) ^B^	10/10 (100%)	1.8	4.0 (0.33) ^BC^	6.9 × 10^3^ (1849) ^CD^	1.6 × 10^4^ (4932) ^BC^
mRNA LS-P2-VP8* **(12 µg)**	13	12/13 (92%)	1.9	3.3 (0.51) ^AB^	9.1 (0.85) ^B^	7.8 (0.72) ^B^	13/13 (100%)	1.7	3.6 (0.37) ^C^	4.5 × 10^3^ (1182) ^D^	8.3 × 10^3^ (2619) ^C^
mRNA LS-P2-VP8* **(30 µg)**	10	9/10 (90%)	2.4	2.5 (0.48) ^B^	9.1 (0.91) ^B^	7.9 (0.83) ^B^	10/10 (100%)	1.7	4.1 (0.43) ^BC^	1.2 × 10^4^ (2335) ^ABC^	2.1 × 10^4^ (5103) ^B^

Note: a. Pigs were immunized three times with an irrelevant mRNA vaccine as control, P2-VP8* protein, or one of two rotavirus mRNA vaccines at 5 days (post-inoculation day [PID] 0), 19 days (PID 14), and 33 days (PID 28) of age. On PID 35, all pigs were orally challenged with 1 × 10^5^ FFU of virulent Wa HRV and monitored for diarrhea and virus shedding for 7 days post-challenge. b. Fecal consistency scores were used to assess diarrhea; scores are defined as 0: solid, 1: pasty, 2: semi-liquid, and 3: liquid. Scores of 2 or higher are considered diarrheic. c. Rotavirus-shedding titers were determined by rotavirus antigen ELISA (detect viral antigen) and CCIF (determine the number of infectious viral particles). If there is no diarrhea or virus shedding, the mean days to onset were assigned as one day after the pigs were euthanized (PCD 8) for statistical analysis. d. Different capital letters indicate significant differences between groups (*n* =  8–13; unadjusted *p*  <  0.05), while shared letters or no letters indicate no significant difference. e. Numbers in parentheses represent the standard error of the mean (SEM). f. Mean peak titer and AUC of virus-shedding titers were log_10_ transformed prior to statistical analysis.

## Data Availability

The authors declare that all relevant data supporting the findings of this study are available within the paper and its Supplementary Materials. Additional information and underlying data are available from the corresponding author upon reasonable request.

## Supplementary Materials

The following supporting information can be downloaded at: https://www.mdpi.com/article/10.3390/vaccines12030260/s1, Supplementary Figure S1. Electropherogram of LS-P2-VP8* P[8] mRNA. Upon production, mRNA size and integrity of the final mRNA product was determined using a 12-capillary Fragment Analyzer (Advanced Analytical) and PROSizeTM 3.0 software (Advanced Analytical). The depicted electropherogram (A) and the virtual gel image (B) of LS-P2-VP8 P[8] mRNA serve as a representative example for the size and integrity determination of final mRNA products. RFU: relative fluorescence units; LM: lower marker. Supplementary Figure S2. Timeline of gnotobiotic pig immunization, challenge and sample collections. Supplementary Figure S3. Interferon-α (IFN-α) levels induced by prime vaccination. Serum was taken 14 hours after the first vaccination and evaluated for systemic IFN-α levels using a commercial ELISA kit. Different letters indicate significant differences between groups (*n* = 8–13; unadjusted *p* < 0.05), while shared letters or no letters indicate no significant difference. Bars indicate means with SEM. Supplementary Figure S4. Correlation matrix displaying relationships between disease burden measurements. Measures of virus shedding (AUC of virus shedding, peak titer, shedding days) and diarrhea (AUC of diarrhea, cumulative fecal score, diarrhea days) were pooled across groups. Individual observations for each animal are denoted by points with the red line denoting the fit from a simple linear regression. Spearman correlations (Corr) with corresponding P-values are listed within each panel. Supplementary Figure S5. Correlations between disease burden and P-type-specific IgG responses. Measures of disease burden (AUC of virus shedding, shedding days, AUC of diarrhea, diarrhea days) and P-type-specific serum IgG responses at PID 28 (A) or PCD 0 (B) were pooled across groups. Individual observations for each animal are denoted by points with the blue line denoting the fit from a simple linear regression. Spearman correlations (rho) with corresponding P-values are listed within each panel. Supplementary Figure S6. Correlations between disease burden and IFN-α responses. Measures of disease burden (AUC of virus shedding, shedding days, AUC of diarrhea, diarrhea days) and serum IFN-α responses induced 14 h post-prime immunization were pooled across groups. Individual observations for each animal are denoted by points with the blue line denoting the fit from a simple linear regression. Spearman correlations (rho) with corresponding P-values are listed within each panel. Supplementary Figure S7. Gating strategy for detection of IFN-γ producing CD4 and CD8 T responses in Gn pigs. Supplementary Figure S8. Total mean numbers and frequencies of CD3+CD4+IFN-γ+ (A) and CD3+CD8+IFN-γ+ (B) in the blood pre-challenge. There were no significant differences according to ordinary one-way ANOVA followed by Dunnett’s multiple comparisons test (*n* = 8–13; * adjusted *p* ≤ 0.05). Bars indicate means with SEM. Supplementary Figure S9. Mean numbers of P[6]-specific ASCs in the tissues of Gn pigs post-challenge. MNCs extracted from post-mortem tissues were evaluated for P-type specific IgG (A) or IgA (B) ASCs using ELISpot assay. Kruskal-Wallis test followed by Dunn’s multiple comparisons test was used for analysis (*n* = 8–13; * *p* ≤ 0.05). Bars indicate means with SEM. ELISpot, enzyme-linked immunosorbent spot; ASC, antibody-secreting cells; MNCs, mononuclear cells. Supplementary Figure S10. Mean numbers of P[4]-specific ASCs in the tissues of Gn pigs post-challenge. MNCs extracted from post-mortem tissues were evaluated for P-type specific IgG (A) or IgA (B) ASCs using ELISpot assay. Kruskal-Wallis test followed by Dunn’s multiple comparisons test was used for analysis (*n* = 8–13; * *p* ≤ 0.05). Bars indicate means with SEM. ELISpot, enzyme-linked immunosorbent spot; ASC, antibody-secreting cells; MNCs, mononuclear cells.
