# Supplementary material for: mRNA-Based Vaccines Are Highly Immunogenic and Confer Protection in the Gnotobiotic Pig Model of Human Rotavirus Diarrhea
Source: Vaccines (Basel). 2024 Mar 1;12(3):260. doi: 10.3390/vaccines12030260 (PMC10974625; doi:10.3390/vaccines12030260)
Supplement: Supplementary file 1 [file vaccines-12-00260-s001.zip › vaccines-2852225-supplementary.pdf]

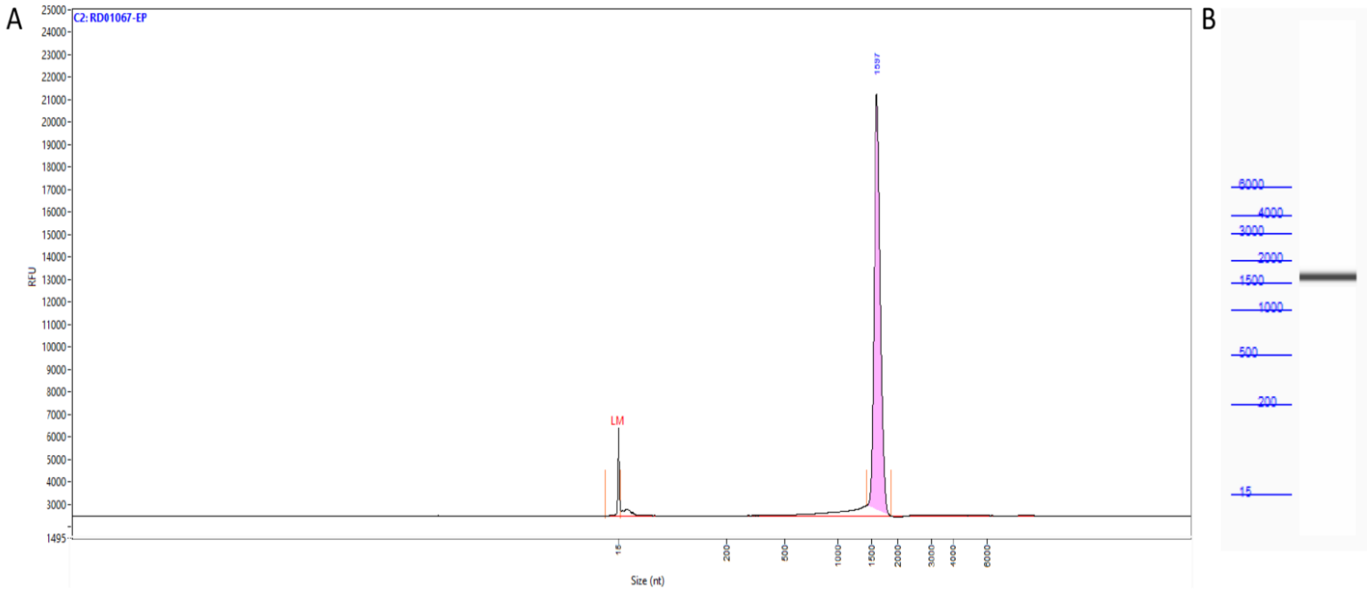


**Supplementary Figure S1.** Electropherogram of LS-P2-VP8* P[8] mRNA. Upon production, mRNA size and integrity of the final mRNA product was determined using a 12-capillary Fragment Analyzer (Advanced Analytical) and PROSizeTM 3.0 software (Advanced Analytical). The depicted electropherogram (A) and the virtual gel image (B) of LS-P2-VP8 P[8] mRNA serve as a representative example for size and integrity determination of final mRNA products. RFU: relative fluorescence units; LM: lower marker.

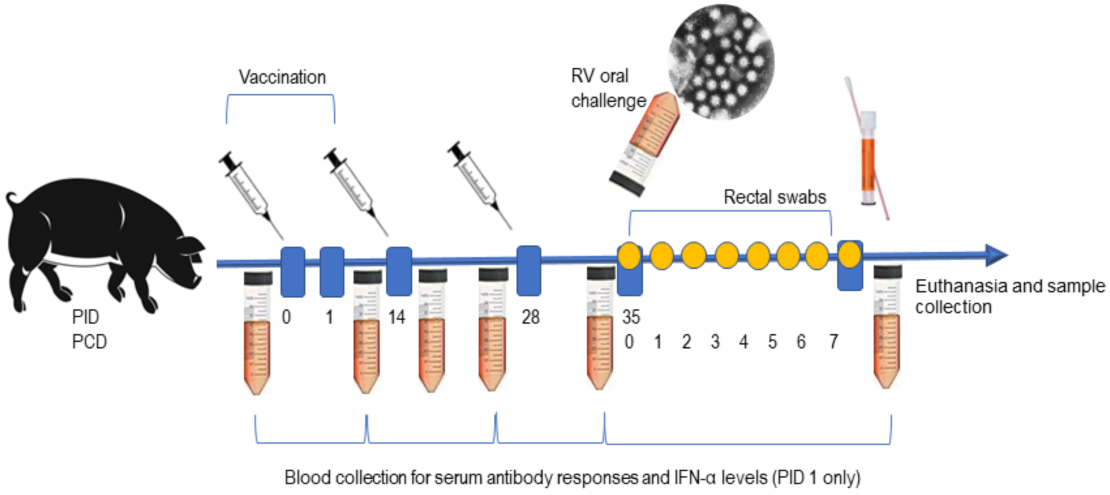


**Supplementary Figure S2.** Timeline of gnotobiotic pig immunization, challenge and sample collections.

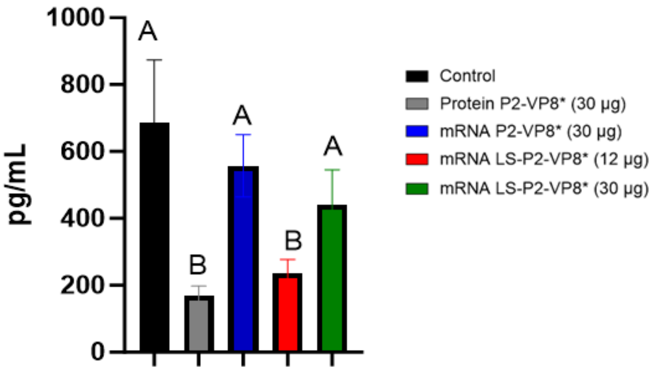


**Supplementary Figure S3.** Interferon-α (IFN-α) levels induced by prime vaccination. Serum was taken 14 hours after the first vaccination and evaluated for systemic IFN-α levels using a commercial ELISA kit. Different letters indicate significant differences between groups (n = 8-13; unadjusted p < 0.05), while shared letters or no letters indicate no significant difference. Bars indicate means with SEM.

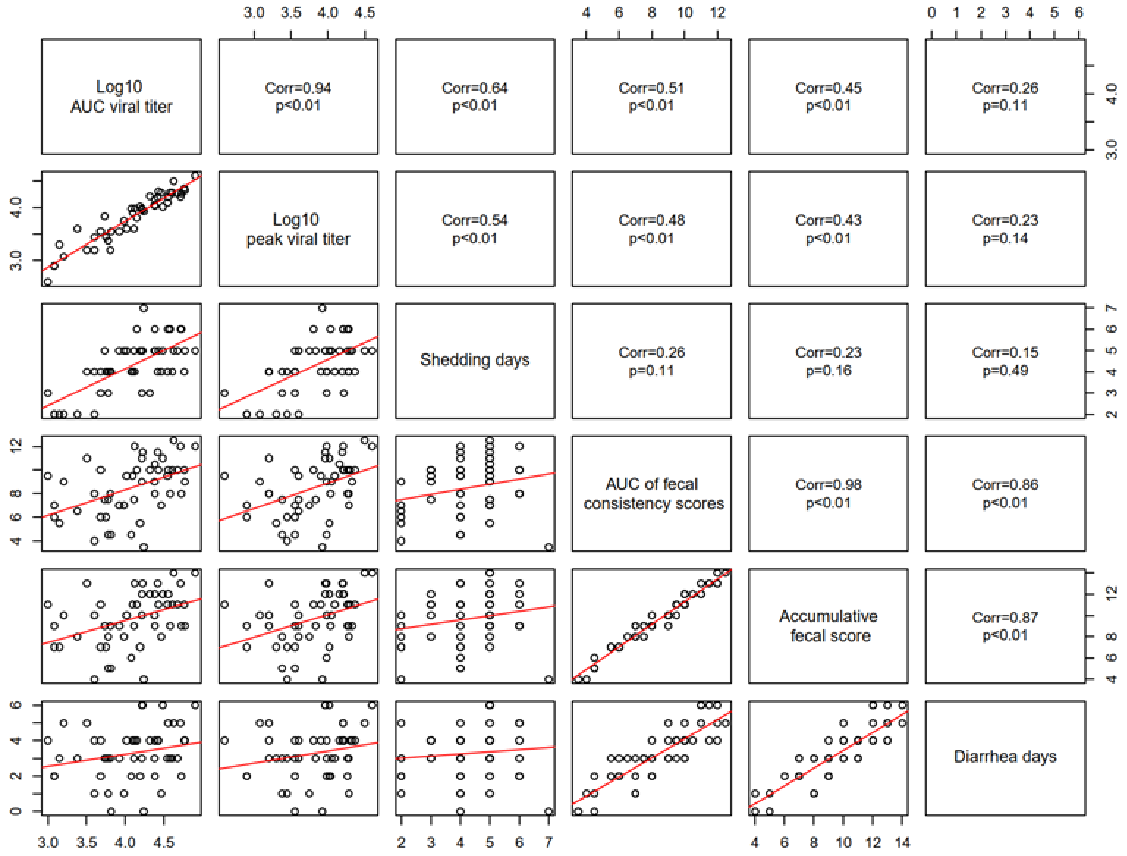


**Supplementary Figure S4.** Correlation matrix displaying relationships between disease burden measurements. Measures of virus shedding (AUC of virus shedding, peak titer, shedding days) and diarrhea (AUC of diarrhea, cumulative fecal score, diarrhea days) were pooled across groups. Individual observations for each animal are denoted by points with the red line denoting the fit from a simple linear regression. Spearman correlations (Corr) with corresponding P-values are listed within each panel.

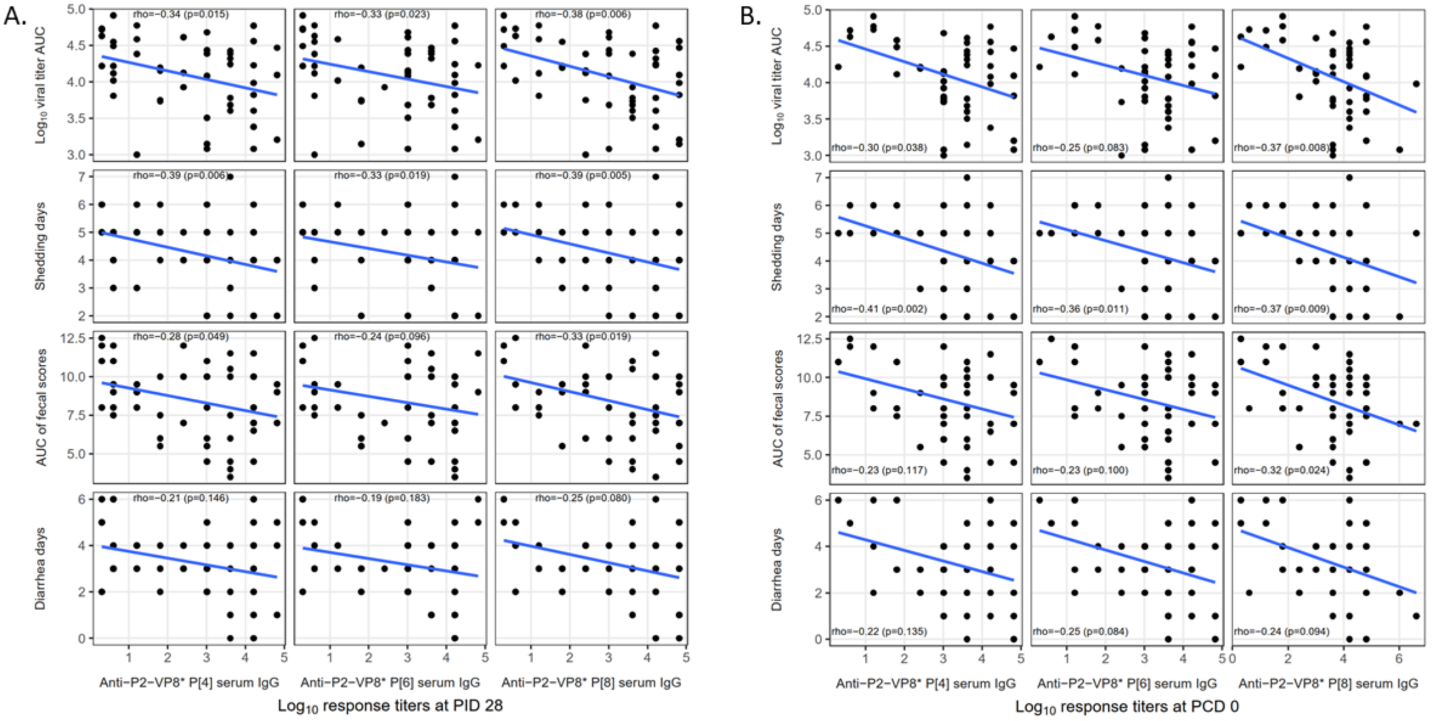


**Supplementary Figure S5.** Correlations between disease burden and P-type-specific IgG responses. Measures of disease burden (AUC of virus shedding, shedding days, AUC of diarrhea, diarrhea days) and P-type-specific serum IgG responses at PID 28 (A) or PCD 0 (B) were pooled across groups. Individual observations for each animal are denoted by points with the blue line denoting the fit from a simple linear regression. Spearman correlations (rho) with corresponding P-values are listed within each panel.

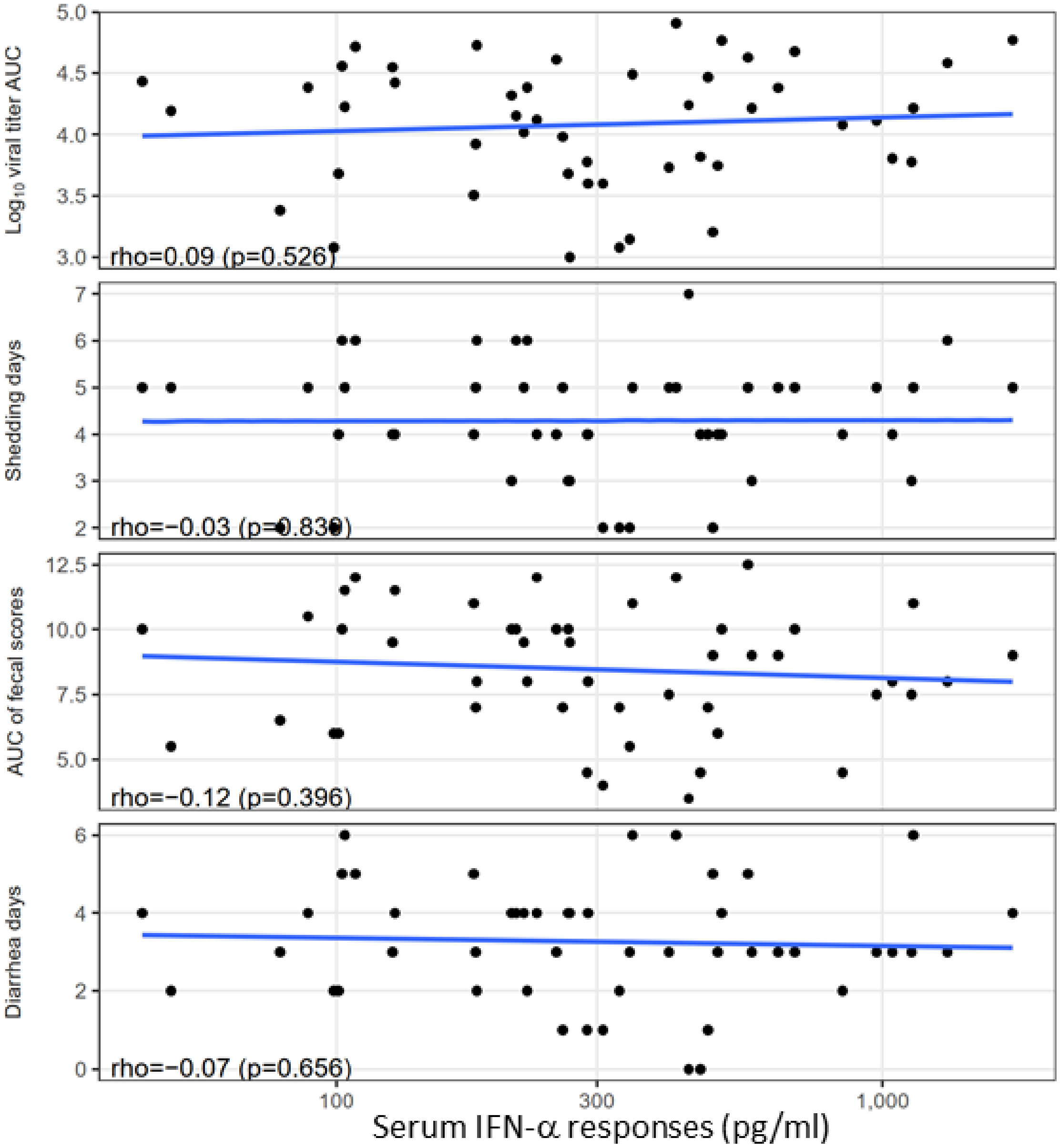


**Supplementary Figure S6.** Correlations between disease burden and IFN-$\alpha$ responses. Measures of disease burden (AUC of virus shedding, shedding days, AUC of diarrhea, diarrhea days) and serum IFN-$\alpha$ responses induced 14 h post-prime immunization were pooled across groups. Individual observations for each animal are denoted by points with the blue line denoting the fit from a simple linear regression. Spearman correlations (rho) with corresponding P-values are listed within each

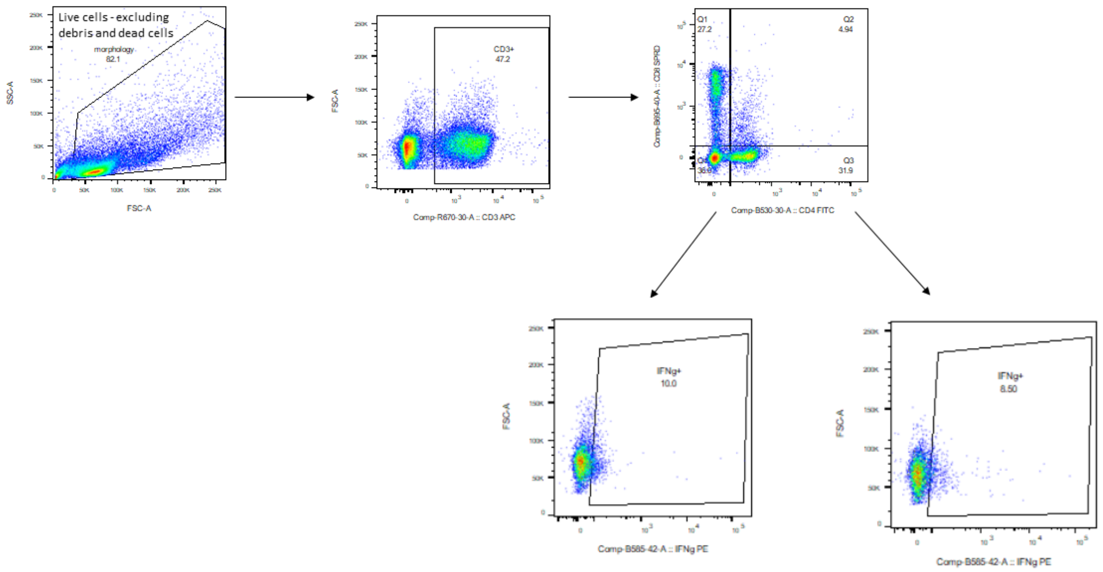


**Supplementary Figure S7.** Gating strategy for detection of IFN-γ producing CD4 and CD8 T responses in Gn pigs.

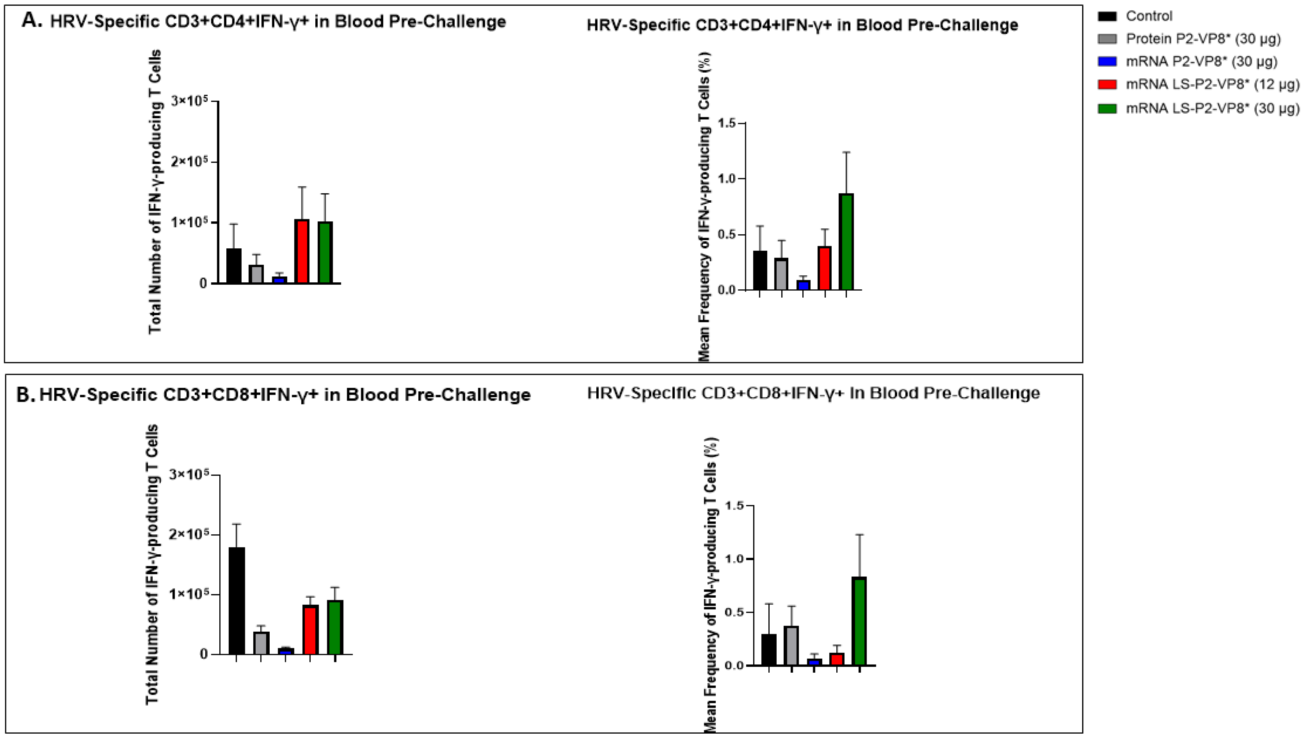


**Supplementary Figure S8.** Total mean numbers and frequencies of CD3+CD4+IFN-γ+ (A) and CD3+CD8+IFN-γ+ (B) in the blood pre-challenge. There were no significant differences according to ordinary one-way ANOVA followed by Dunnett's multiple comparisons test (n=8-13; * adjusted $p \leq 0.05$). Bars indicate means with SEM.

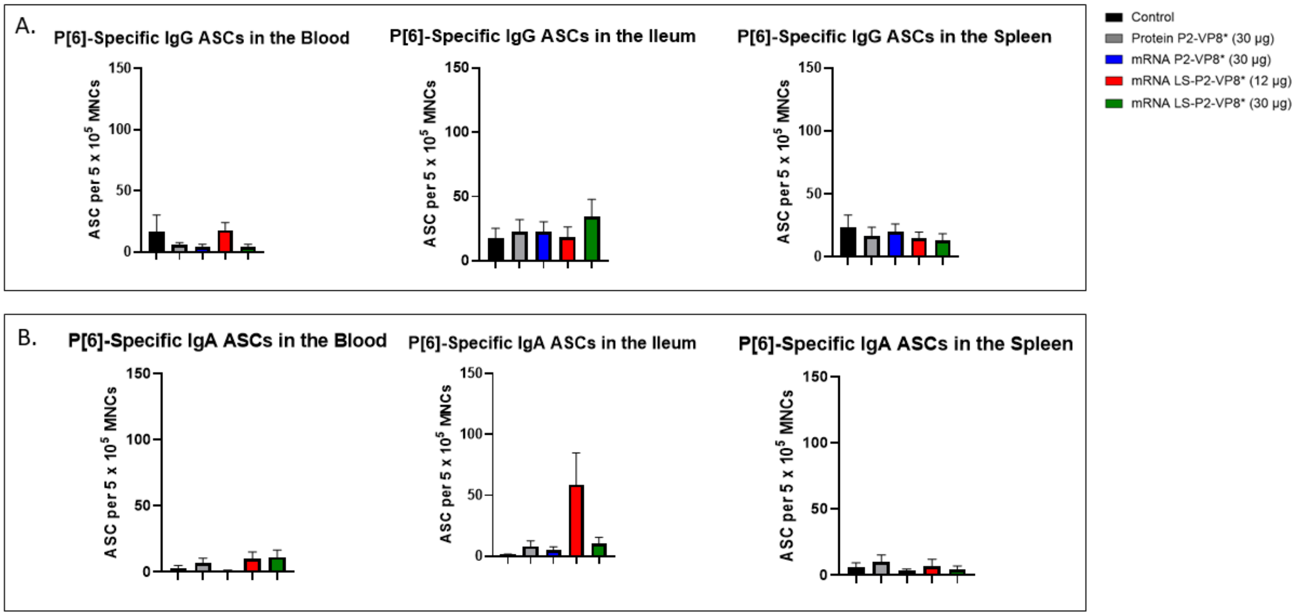


**Supplementary Figure S9.** Mean numbers of P[6]-specific ASCs in the tissues of Gn pigs post-challenge. MNCs extracted from post-mortem tissues were evaluated for P-type specific IgG (A) or IgA (B) ASCs using ELISpot assay. Kruskal-Wallis test followed by Dunn's multiple comparisons test was used for analysis (n=8-13; * $p \leq 0.05$). Bars indicate means with SEM. ELISpot, enzyme-linked immunosorbent spot; ASC, antibody-secreting cells; MNCs, mononuclear cells.

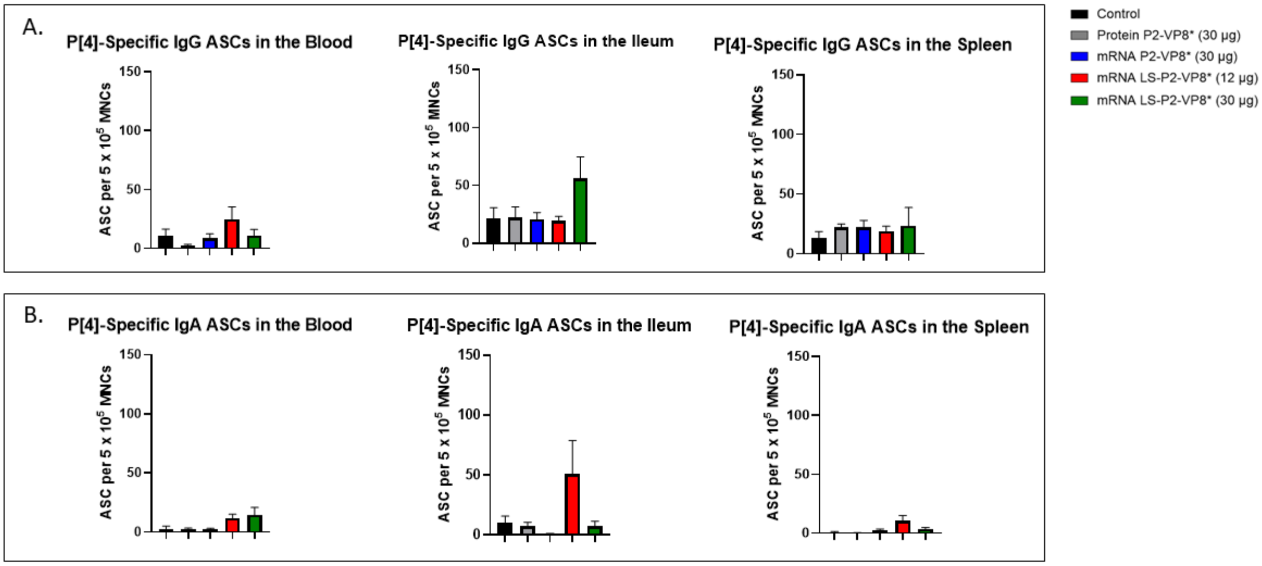


**Supplementary Figure S10.** Mean numbers of P[4]-specific ASCs in the tissues of Gn pigs post-challenge. MNCs extracted from post-mortem tissues were evaluated for P-type specific IgG (A) or IgA (B) ASCs using ELISpot assay. Kruskal-Wallis test followed by Dunn's multiple comparisons test was used for analysis (n=8-13; * $p \leq 0.05$). Bars indicate means with SEM. ELISpot, enzyme-linked immunosorbent spot; ASC, antibody-secreting cells; MNCs, mononuclear cells.
